# Use of 3D printing models for donor tooth extraction in autotransplantation cases

**DOI:** 10.1186/s12903-024-03864-z

**Published:** 2024-02-04

**Authors:** Rui Hou, Xiaoyong Hui, Guangjie Xu, Yongqing Li, Xia Yang, Jie Xu, Yanli Liu, Minghui Zhu, Qinglin Zhu, Yu Sun

**Affiliations:** 1 Department of Oral & Maxillofacial Surgery, School of Stomatology, State Key Laboratory of Oral & Maxillofacial Reconstruction and Regeneration & National Clinical Research Center for Oral Diseases & Shaanxi Clinical Research Center for Oral Diseases, The Fourth Military Medical University, Xi’an, 710032 China; 2 Rocket Army Guangzhou Special Service Sanitarium, Guangzhou, 510515 China; 3 Department of Stomatology, Naval Hospital of Eastern Theater, Zhoushan, 316000 China; 4 Department of Periodontology, School of Stomatology, State Key Laboratory of Oral & Maxillofacial Reconstruction and Regeneration & National Clinical Research Center for Oral Diseases & Shaanxi International Joint Research Center for Oral Diseases, The Fourth Military Medical University, Xi’an, 710032 China; 5 Department of General Dentistry and Emergency, School of Stomatology, State Key Laboratory of Oral & Maxillofacial Reconstruction and Regeneration & National Clinical Research Center for Oral Diseases & Shaanxi International Joint Research Center for Oral Diseases, The Fourth Military Medical University, Xi’an, 710032 China; 6 Department of Operative Dentistry and Endodontics, School of Stomatology, State Key Laboratory of Oral & Maxillofacial Reconstruction and Regeneration & National Clinical Research Center for Oral Diseases & Shaanxi Key Laboratory of Stomatology, The Fourth Military Medical University, Xi’an, 710032 China; 7 Medical Research and Development Center, Shaanxi Huikang Bio-Tech Co.,Ltd, Xi’an, 710054 China

**Keywords:** Autotransplantation, Extraction, Donor tooth, 3D printing technology

## Abstract

**Objective:**

To clarify whether the 3D printing model has auxiliary functions in toto extraction of donor tooth in autotransplantation cases.

**Methods:**

Two hundred and sixty patients who would have operation of ATT were divided into two groups. In group 1, determination of the tooth extraction in toto was predicted only according to the clinical and imaging examination. In group 2, the prediction was performed according to the clinical and imaging examination as well as the 3D model of donor tooth pre-extraction. A prespctive clinical study was designed on intra-group comparison between the predicted and actual donor teeth situation when extraction in cases of ATT. The consistent rate for the predicted results and the actual results were compared with the two groups.

**Results:**

A remarkable difference was observed between the predicted results and the actual results of tooth positions and root numbers in group without model (*p* < 0,05). The consistency rate of the model group (94.62%) was significantly higher than that of non 3D model group (86.15%) (*p* = 0.034).

**Conclusion:**

The 3D printing model for the donor tooth is helpful for dentists to predict the accuracy of toto extraction of donor teeth in autotransplantation cases.

Autotransplantation of teeth (ATT) is the surgical transposition of a donor tooth from a donor site to a recipient socket within the same individual, where teeth are absent due to hypodontia, trauma or pathological loss. With high success and survival rate, it has been considered as the most cost-effective biological alternative to nonrestorable tooth replacement comparing with dental implantation especially in the young and the grown-up patients [1–9]. Meanwhile, autotransplanted tooth has the potential to induce the growth of socket bone, maintain the height of socket bone and gum margin, protect the integrity of the dentition, and also enable to be mobilizable via orthodontics force [10–13].

Nowadays 3D printing technology is commonly used on ATT. Various 3D printing materials were performed via fused deposition modelling (FDM) or polymer injection technique to prepare donor tooth models [14–22], with the advantage of shortening the extraoral time [15, 16], and reducing the periodontal ligament damage to donor teeth [17, 18], as well as accurately preparing the alveolar socket [19, 20], moreover promoting post-surgical healing of soft and hard tissue in the recipient area [21, 22].

In toto extraction of donor tooth plays an important role in the prognosis of ATT because the complete donor tooth can be directly transplanted into the socket of the recipient area. Once the root fracture occurs, it is necessary to prepare and fill the root tip retrograde during the operation to prevent the root tip from retrograde infection, which may increase the donor teeth in vitro time and the operational trauma risk of the periodontal ligament of donor teeth. They are the key to the success of autologous tooth transplantation.

However, the most concern of 3D printing technology was focusing on intraoperative application of the donor tooth model, rather than exploring the application in the process of the whole donor tooth extraction. In addition, neither oral surgeons nor general practitioners have reported their cognition of the model assisted tooth extraction. They usually predict whether whole donor teeth can be extracted in toto only depends on clinical and imaging examinations pre-autotransplantation, but the absence of stereoscopic imaging of the donor teeth on numbers, shape, and root bifurcation angle may result in inaccurate preoperative evaluation, inadequate instruments preparation, Insufficient use of operating skills, even the failure of whole donor teeth extraction.

Therefore, we performed 3D printing model of donor teeth pre-extraction, and designed a prespective clinical study to clarify its auxiliary effect on the whole donor teeth extraction.

## Methods

This study was reviewed and approved by the medical ethics committee School of Stomatology the Fourth Military Medical University (Approval No.: IRB-REV-2,018,064 and 2,020,027). The clinical trial had been registered in Chinese Clinical Trial Registry (http://www.chictr.org.cn/index.aspx). The public title is Medical records based study for the application of 3D printing technology in autotransplantation of teeth. The registration number is ChiCTR2100049688 (Registered 8 August 2021 - Retrospectively registered).

### Preparation of 3D printing model

According to the different densities of teeth and jaws in cone-beam CT before operation, technicians segmented the three-dimensional data of donor teeth and exported them to the printing software (Cural, V15.06) of a 3D printer (Aurora Wall A9 printer, Shenzhen). The nozzle diameter of the printer is 0.4 mm, the printing layer thickness is 0.05 mm, the printing material is linear polylactic acid with a diameter of 1.75 mm, and the printing speed is 30 mm/s. Finally, 800 mg/L ethylene oxide was performed to sterilize the model at 55 ~ 60℃ for 6 h for following study.

### Surgical procedure

The routine operation steps of ATT was as follows: The affected tooth was pulled out under local anesthesia firstly. Usually, the donor tooth was extracted in toto and immediately transplanted into the socket of the recipient area. While, in most cases, the donor teeth was not completely in place, so it was necessary to prepare the socket or adjust the occlusal high point. If there was a 3D printing model, the repeatedly attempts to put the model into the socket for trial implantation were made with judging and grinding the bottom wall as well as the side wall of the socket until the donor tooth can get the ideal position. Then the surgeon pulled out the donor tooth and transplanted it into the socket of the receiving area with reference to the direction and angle of the model position. Finally, Splint Woven (RTD, France) was used to fix the transplanted teeth with the adjacent teeth elastically.

### Clinical research on the effect of 3D technology in the extraction of donor teeth

All the research results have been authorized by 260 patients (16–65 years, unisex) undergoing ATT from September 2018 to February 2021 in the Department of Oral & Maxillofacial surgery School of Stomatology the Fourth Military Medical University.

**Fig. 1:**
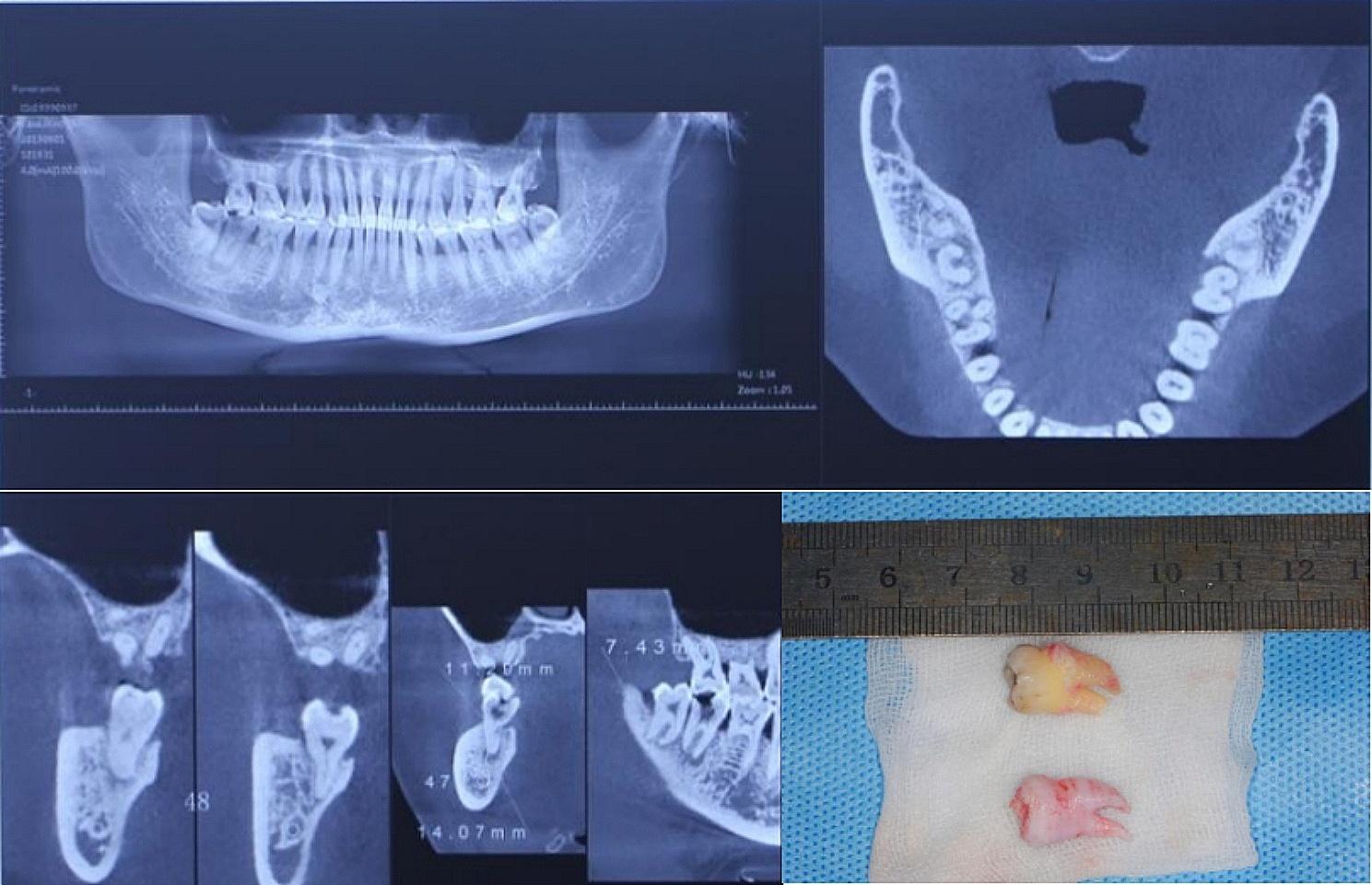
Preoperative CBCT and 3D printing model of donor tooth and the actual tooth in a ATT case. The CBCT showed the affected tooth (mandible second molar) had a residual crown and obvious tip shadow. The donor tooth(mandible third molar) had good shape for crown and double roots. The treatment plan was to transplant the mandible third molar to replace the mandible second molar; 3D printing model showed that the bending angle of distal buccal root of donor tooth was larger, and the actual tooth showed that the distal buccal root was fractured for about 1 mm

**Fig. 2:**
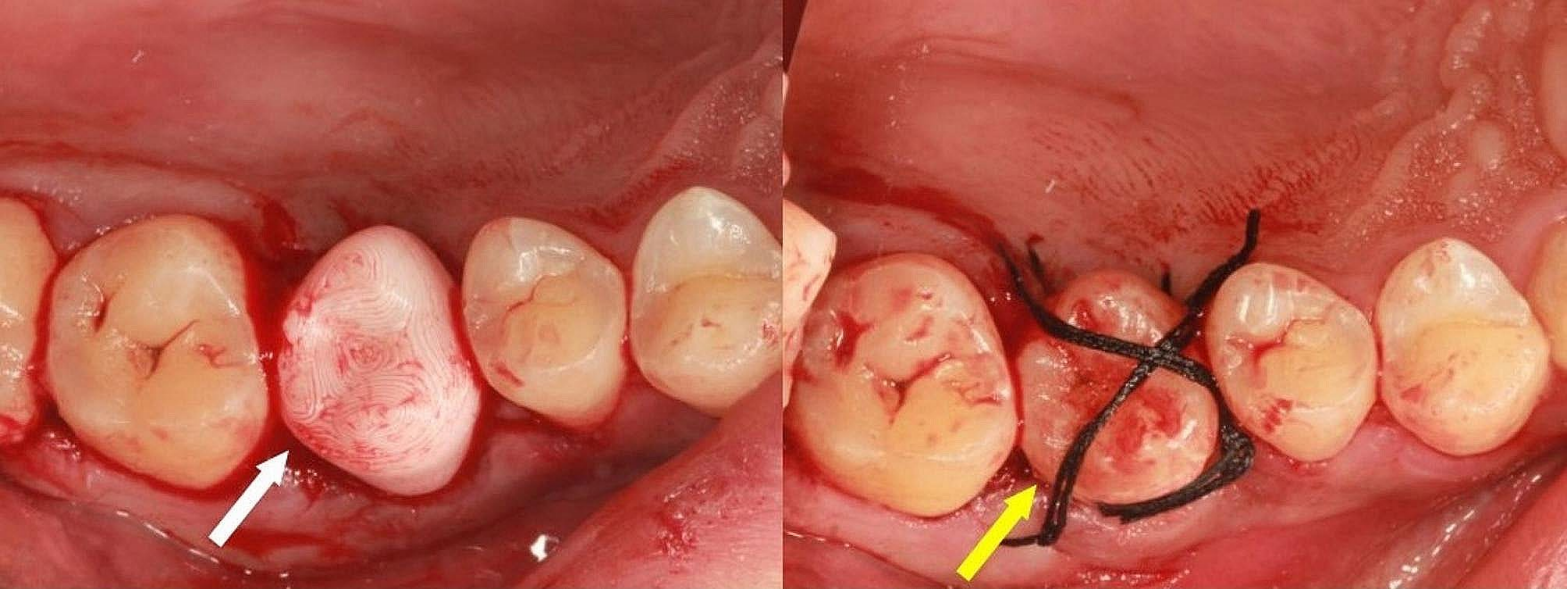
Intraoral image showed the printing model and transplanted tooth were in place during autotransplantation. The white arrow points to the printing model, while the yellow arrow points to the transplanted tooth

They were randomly divided into 2 groups (*N* = 130). In group 1, an oral surgeon predicted whether the donor tooth could be extraction in toto or not according to the clinical and imaging examination on the donor tooth position, number, root shape and impacted condition pre-operation. Then the extraction operation was carried out by the same surgeon. The same procedure was performed on group 2, the only difference was that the prediction was made according to the clinical and imaging examination as well as the 3D model of donor tooth pre-extraction (Fig. 1). Then the ATT operation was completed with the help of the model. (Fig. 2). The integrity of extracting donor teeth was judged and recorded by another dentist compared with the predicted results.

Firstly, intra-group comparison between the predicted and the actual situation of the donor teeth in different cases was done. Then, the consistent rate of the predicted results and the actual results were compared with the two groups.

The consistency rate between the predicted results and the actual results was calculated using the following formula:

$$\eqalign{& {\rm{Consistency}}\,{\rm{rate}} = \cr & \frac{{{\rm{the}}\,{\rm{number}}\,{\rm{of}}\,{\rm{patients}}{\rm{with}}\,{\rm{the}}\,{\rm{predicted}}\,{\rm{results}}\,{\rm{consistent}}\,{\rm{with}}\,{\rm{the}}\,{\rm{actual}}\,{\rm{results}}}}{{{\rm{the}}\,{\rm{total}}{\rm{number}}\,{\rm{of}}\,{\rm{patients}}}} \cr & \times 100\% .}$$

### Statistical methods

SPSS23.0 software (https://www.ibm.com/cn-zh/analytics/products) was used in the study. Consistency test and chi square (χ²) test were performed to compare the difference in consistency rate between the two preoperative predicted results and the actual results. The difference in toto extraction of donor teeth was compared between the two groups using χ² test. *p* < 0.05 indicated the statistical significance, while, *p* < 0.01 was as extremely significant difference.

## Results

Table 1 showed the intra-group comparison results compared the predicted with the actual situation of the donor teeth in different cases. In group 1, remarkable difference was observed between the predicted results and the actual results of tooth positions and root numbers, but not in the impacted conditions. However, no significant difference was found in group 2.

Meanwhile, by comparing the consistency rates between group1 and group 2 (Table 2), the consistency rates (94.62%) dependent on the clinical and imaging examination with 3D model was significantly higher than that according to the prediction via examination without 3D model (86.15%) (*p* = 0.034).

**Table 1:** Intra-group comparison results between the predicted and the actual situation of the donor teeth in different cases (number of consistent/ number of inconsistent)

Group	Number of teeth	Tooth position		Root number			Impacted position		
		Maxillary teeth	Mandibular teeth	Single rooted teeth	Double rooted teeth	Triple or more rooted teeth	High impacted	Median impacted	Low impacted
Group1	112/18	64/10	48/8	48/3	43/7	21/8	52/6	39/8	21/4
χ²		7.238		7.302			1.090		
*p*		0.010		0.026			0.580		
Group2	123/7	71/3	52/4	49/2	48/2	26/3	56/2	44/3	23/2
χ²		0.597		1.803			0.854		
*p*		0.440		0.406			0.652		

**Table 2:** The comparative results of donor teeth extraction between two groups

Group	Number of Consistent	Number of Inconsistent	Percentage of complete extraction	Total number	χ²	*p*
Group1	112	18	86.15	130	5.355	0.034
Group2	123	7	94.62	130		

## Discussion

Many studies have found that 3D model is helpful to reduce surgical trauma and achieve a good prognosis [14–22]. For the model material, PLA has the advantages of good toughness, well stability, excellent water tightness and high durability. It will not produce toxicity or side effects during manufacturing and finally degrade into harmless lactic acid. In addition, compared with other model materials, the manufacturing method of PLA is simpler than cobalt-chromium alloy [20, 23], the environmental protection is better than light-cured UDMA material [22] and acrylic resin [19] material, and the manufacturing cost is the lowest.

However, most literature only focus on the intraoperative application of 3D model instead of exploring its preoperative application and function in donor tooth extraction [14–22]. In order to understand the effect of 3D model on ATT donor tooth extraction, a clinical study was explored in the present study.

The slinical study results indicate that 3D model can provide information beyond what was traditionally gathered in clinical and imaging examinations, so as to improve the consistency rate between predicted results and actual results.

Furthermore, if the donor tooth is a highly impacted maxillary single rooted tooth, the consistency rate between the predicted results and the actual results is relatively high regardless of the model prediction. On the contrary, if the donor tooth is a median or low impacted mandibular tooth with two or more roots, the consistency rate is relatively low. The corresponding reasons are as follows: (1) The maxillary bone is more osteoporotic. The impaction state of maxillary impacted teeth is simpler than that of mandibular impacted teeth. Most of them have fully erupted teeth, so it is easy to predict. (2) The single rooted teeth are usually conical, due to numerous roots of multiple rooted teeth and big bifurcation in most of them, it is not uncommon to see malformed micro-roots that are difficult to be recognized by CT, so the former is easy to predict. (3) The elimination of resistance of mucous membranes mainly focused on high impacted donor extraction, which is an easy process of toto extraction; whereas, the elimination of resistance of bone and adjacent teeth is a difficult surgical process in low impacted donor tooth extraction, leading to the incomplete tooth extraction. Both of them are easy to predict preoperative with high consistency rate. However, the situation of middle impacted donor teeth is complex, and the consistency rate between prediction and actual results is the lowest.

Table 2 shows that with the help of the 3D model, we can intuitively observe the shapes and integrities of the various surfaces of the tooth crown, the number, shape, length and developmental status of the tooth root, the angle of the root bifurcation as well as the excessively curved and slender root tip in the donor tooth. We can also find adjacent caries, weak part of hard tissue, and malformed micro-roots that are missed or incompletely demonstrated on CT, so as to supplement the insufficient imaging examination. At the same time, it also reminds both general practitioners and oral surgeons that once it is expected that the donor teeth cannot be extracted in toto, the surgical plan should be designed after root fracture, and prepare retrograde root canal filling instruments before operation, and avoid improper force and blind operation during the operation. For the actual failure to extract the donor tooth in toto, it is necessary to summarize the reasons in time and make later improvement.

Further comparative study resulted that the prediction coincidence rate of donor tooth extraction in the model group is statistically significant higher than that in no model group. This is a new function of the 3D model of donor teeth that we discover and summarize in ATT operation. This also suggests that we can continue to look for the role of the model in postoperative fixation and root canal therapy, and further explore the role of the model in preparing alveolar fossa during operation.

In the present study, the strengths of using 3D model provide dentists with further evidence for tooth extraction prediction and help general practitioners to improve pre-operative preparation of tooth extraction, so as to facilitate the subsequent transplantation surgery. However, there are also some limitations, such as the need for professionals to make the 3D model, the necessity for additional time to make model, and the increase in patient medical expenses.

## Conclusion

The clinical application of 3D printing technology has been widely promoted. However, the application of 3D model before extraction of donor tooth in ATT has not been paid attention to. It should be further recognized that the accurate 3D model can help dentists to carry out preoperative prediction on extraction of donor tooth.

## Data Availability

As the related follow-up research is still going on, once the data is made public, it may cause data abuse, so the datasets generated and/or analysed during the current study are not publicly available, but are available from the corresponding author on reasonable request.

## References

[1] Tsukiboshi M (2002). Autotransplantation of teeth: requirements for predictable success[J]. Dent Traumatol.

[2] Hou R, Xu GJ, Hui XY (2018). Clinical analysis of 300 cases of autotransplantation[J]. China J Oral Maxillofac Surg.

[3] Andreasen JO, Paulsen HU, Yu Z (1990). A long-term study of 370 autotransplanted premolars. Part IV. Root development subsequent to transplantation[J]. Eur J Orthod.

[4] Kim S, Shin SJ, Park JW (2016). Long-Term Stability of Autotransplanted premolars as a Substitute for molars in Adults[J]. J Endod.

[5] Jang Y, Choi YJ, Lee S (2016). Prognostic factors for clinical outcomes in Autotransplantation of Teeth with Complete Root formation: Survival Analysis for up to 12 Years[J]. J Endod.

[6] Plakwicz P, Wojtowicz A, Czochrowska EM (2013). Survival and success rates of autotransplanted premolars: a prospective study of the protocol for developing teeth[J]. Am J Orthod Dentofacial Orthop.

[7] Verweij JP, Toxopeus EE, Fiocco M (2016). Success and survival of autotransplanted premolars and molars during short-term clinical follow-up[J]. J Clin Periodontol.

[8] Rohof ECM, Kerdijk W, Jansma J (2018). Autotransplantation of teeth with incomplete root formation: a systematic review and meta-analysis[J]. Clin Oral Investig.

[9] Machado LA, Do Nascimento RR, Ferreira DM, T P (2016). Long-term prognosis of tooth autotransplantation: a systematic review and meta-analysis[J]. Int J Oral Maxillofac Surg.

[10] Mertens B, Boukari A, Tenenbaum H (2016). Long-term follow up of post-surgical tooth autotransplantation: a retrospective study[J]. J Investig Clin Dent.

[11] Andreasen JO, Paulsen HU, Yu Z (1990). A long-term study of 370 autotransplanted premolars. Part I. Surgical procedures and standardized techniques for monitoring healing[J]. Eur J Orthod.

[12] Andreasen JO, Paulsen HU, Yu Z (1990). A long-term study of 370 autotransplanted premolars. Part II. Tooth survival and pulp healing subsequent to transplantation[J]. Eur J Orthod.

[13] Andreasen JO, Paulsen HU, Yu Z (1990). A long-term study of 370 autotransplanted premolars. Part III. Periodontal healing subsequent to transplantation[J]. Eur J Orthod.

[14] Verweij JP, Jongkees FA, Anssari Moin D (2017). Autotransplantation of teeth using computer-aided rapid prototyping of a three-dimensional replica of the donor tooth: a systematic literature review[J]. Int J Oral Maxillofac Surg.

[15] Lee SJ, Kim E (2012). Minimizing the extra-oral time in autogeneous tooth transplantation: use of computer-aided rapid prototyping (CARP) as a duplicate model tooth[J]. Restor Dent Endod.

[16] Hou R, Hui XY, Xu GJ (2020). Clinical observation of three-dimensional printing model of donor teeth in perioperative period of autotransplantation of tooth[J]. Chin J Stomatol.

[17] Richard RJC, Andrew G, Jeremy N (2017). A 3D printed surgical analogue to reduce donor tooth trauma during autotransplantation. J Orthod.

[18] Kim K, Choi H, Pang N (2019). Clinical application of 3D technology for tooth autotransplantation: a case report[J]. Aust Endod J.

[19] Malka A, Dafna S, Salo K (2018). Computerized three-dimensional design for accurate orienting and dimensioning artificial dental socket for tooth autotransplantation[J]. Quintessence Int.

[20] Wicher JM, Johan J, Konstantina D (2016). Computer-aided planning and surgical guiding system fabrication in premolar autotransplantation: a 12-month follow up[J]. Dent Traumatol.

[21] Abella F, Ribas F, Roig M (2018). Outcome of autotransplantation of mature third molars using 3-dimensional-printed guiding templates and donor tooth Replicas[J]. J Endod.

[22] Honda M, Uehara H, Uehara T (2010). Use of a replica graft tooth for evaluation before autotransplantation of a tooth. A CAD/CAM model produced using dental-cone-beam computed tomography[J]. Int J Oral Maxillofac Surg.

[23] Shahbazian M, Jacobs R, Wyatt J (2010). Accuracy and surgical feasibility of a CBCT-based stereolithographic surgical guide aiding autotransplantation of teeth: in vitro validation[J]. J Oral Rehabil.

